# Comparison between multi-walled carbon nanotubes and titanium dioxide nanoparticles as additives on performance of turbine meter oil nano lubricant

**DOI:** 10.1038/s41598-021-90625-5

**Published:** 2021-05-26

**Authors:** Hadi Pourpasha, Saeed Zeinali Heris, Yaghob Mohammadfam

**Affiliations:** Faculty of Chemical and Petroleum Engineering, University of Tabriz, Tabriz, Iran

**Keywords:** Energy science and technology, Engineering, Materials science, Nanoscience and technology

## Abstract

This research aims of compare the impact of the mass fraction of multi-walled carbon nanotubes (MWCNTs) and titanium dioxide (TiO_2_) nano additive on the tribological and thermophysical attributes of turbine meter oil. These attributes include the average friction coefficient, pressure drop, wear, flash point, pour point, relative viscosity, kinematics viscosity, and viscosity index. The pressure drops and the average friction coefficient inside the copper tube were simulated and compared with experimental results. In this study, for the synthesis of nano lubricants from turbine meter oil as a pure fluid and from MWCNTs and TiO_2_ as nano additives in the mass fraction of 0.05, 0.1, 0.2, 0.3, and 0.4 wt.% and from oleic acid and Triton x100 as surfactants were utilized. The results illustrated that the wear depth of copper pins in the presence of nano lubricant with 0.4 wt.% of MWCNTs and 0.1 wt.% TiO_2_ was improved by 88.26% and 71.43%, respectively. Increasing 0.3 wt.% of TiO_2_ and MWCNTs into the oil caused to improvement in viscosity index. The simulation data and experimental data for the pressure drop were closer together and indicated a minor error that the maximum error is less than 10%.

## Introduction

The lubrication systems have essential duties in moving mechanical equipment. Since much of the mechanical damage and loss of energy is due to wear and friction, improving the tribological properties of the lubricants used in this equipment is of particular importance, and it is necessary to conduct extensive research in this field. Because almost all traditional lubricants have reached the threshold point of enhancing base oils, a new type of lubricant needs to develop to meet the lubrication requirements of mechanical equipment^1–3^. Nano lubricants are a solution to this challenge and have received much attention in recent years; studies have indicated that adding small amounts of nano additives (about 0.2–2%) to the base fluid can improve its tribological properties^4–7^. Various types of nanoparticles, such as carbon-based materials (including carbon nanotubes and graphene nanoparticles)^8–10^, metal oxides (including titanium dioxide, cobalt oxide, zinc oxide, and iron oxide nanoparticles)^11–13^, and metals (including Al, Cu, Fe, Ni, and Ag), have been used as additives^14–16^. The nanoparticles have unique features due to their high specific surface area and smaller size than other materials. Also, potential advantages of nanoparticles such as low reactivity with other additives, film formation on various surfaces, insolubility in non-polar oils, durability, and withstand high-temperature conditions has led nanoparticles to be interested as additives to improve tribological attributes of oil^17^. However, the principal challenge with the use of nanoparticles is their dispersion and long-term stability in the base fluids. Nanoparticles are highly prone to aggregation because of the strong van der Waals force, which causes the nanoparticles to precipitate and become unstable. Hence, prepare a stable dispersion of nano additives in base fluids is of particular significance. So far, various types of physical and chemical techniques, such as the addition of stabilizing agents, ultrasonic agitation, surface modifications, and mechanical stirring, have been used to prepare a stable nano lubricant, which is very beneficial for excellent lubrication performance^18–20^. TiO_2_ nanoparticles are one of the additives that have been highly regarded for lubrication applications because of their unique attributes such as wear resistance, environmental compatibility, high specific surface area, superior load, and friction attributes. Examples of studies examining the influence of TiO_2_ nano additives on the tribological attributes of lubricants include the following studies. Kao and Lin^21^ investigated the impact of TiO_2_ nano additives on the tribological features of paraffinic lubricant. They used a reciprocating sliding tester to test the wear and friction of the base oil and the nano lubricant. Also, they reported that the friction coefficient achieved for the nano lubricant was less than the value obtained for the base oil, and nanoparticles provide surface repair, rolling function as lubricants. Therefore, spherical TiO_2_ nanoparticles are a good choice for tribological and lubrication applications in the mechanical industry. Sabareesh et al.^22^ examined the impact of TiO_2_ nano additives on the tribological behavior of mineral lubricants. Their results revealed that by increasing the mass fraction of nanoparticles, the viscosity of the nano lubricants rises, which decreases the friction coefficient. Ingole et al.^23^ used TiO_2_ nano additive to improve the tribological performance of the oil and found that increasing nano additives significantly reduced the friction coefficient. Also, they illustrated that increasing nano additives to more than 2 wt.%, reduces the stability of the nanoparticles and increases the friction coefficient. Ali et al.^24^ investigated the effect of TiO_2_, Al_2_O_3_ nanoparticles on the tribological characteristics of engine oil. Their results showed that adding nanoparticles as nano lubricant additives in the base fluid, the wear, power losses, and friction coefficient are decreasing. They also found that adding nanoparticles could improve fuel consumption and reduce energy consumption in automotive engines. Alghani et al.^25^ examined the enhancement of the tribological performance of nano lubricants containing TiO_2_ and graphene nanoparticles. They reported that the nano lubricant containing 0.2 wt.% of graphene and 0.4 wt.% of TiO_2_ provided the best performance with a reduction in specific wear rate and friction coefficient, 15.78 and 38.83, respectively. Hong et al.^26^ investigated the lubrication and dispersion performance of TiO_2_ nanoparticles modified with polyphenol derivatives and unmodified nanoparticles in two base oils, commercial engine oil and polyalphaolefin. The results of their analysis showed that the surface modification of nanoparticles by producing thick tribofilms and strengthening the mechanical properties of the surface helps them to reduce more friction than unmodified nanoparticles. Sharma et al.^27^ reported that TiO_2_ nano additive could provide promising lower friction coefficients and antiwear attributes when used as nano additives in the pure lubricant. Single-walled and multiwalled carbon nanotubes are considered one-dimensional nanomaterials due to their large surface area, which provides a unique combination of chemical, physical, mechanical, and biological properties^28,29^. The chemistry of carbon nanotubes (CNTs) has attracted significant interest in recent years^30,31^. CNTs are another nanomaterial that has always been of interest to researchers due to their anti-wear attributes and high thermal and electrical conductivity. Many researchers have been evaluated the influence of CNTs on the rheological and tribological features of base oils. Based on their results, CNTs are recognized as the best additive to improve the tribological performance of pure oils. Vakilinezhaad et al.^32^ experimentally and theoretically studied the impact of CNTs on the viscosity index of the base fluid. They reported that increasing nano additives to the base oil improves the viscosity index by 14%. Bhaumik et al.^33^ found that adding multi walled carbon nanotubes (MWCNTs) to mineral oil improves the antiwear attributes and load capacity of the pure oil. They reported that mineral oil containing 0.05 wt.% of nano additives reduced wear by 70–75%. Cornelio et al.^34^ reported that increasing of CNTs to pure lubricants causes to improvement in the friction coefficient and wear rate. Khalil et al.^35^ studied the impact of CNTs with the different mass fractions on the tribological property of two base oils (paraffinic mineral and Mobil gear 627 oils). They used a four-ball tribometer to perform wear and friction experiments. Their results showed that increasing of CNTs to the pure lubricant reduced the wear by 39% and 68% in nano lubricant compared with base paraffinic mineral and Mobil gear 627 oils, respectively. Salah et al.^36^ analyzed the tribological performance of various base oils containing CNTs as lubricating additives. They proved that the nano lubricants were containing 0.1 wt.% CNTs reduce the coefficient of friction compared to the pure fluid by 20%. Gao et al.^37^ analyzed the dispersing properties, molecular structures, and physicochemical properties of six types of surfactants, also investigated the tribological properties of CNTs nanofluid with different surfactants. They found that among the six surfactants, nanofluids containing APE-10 showed the optimal dispersion and tribological properties. Li et al.^38^ studied the impact of CNT, graphene, and fullerene on the friction coefficient of the linear alpha-olefin. They reported that graphene and fullerene to the base oil improved the friction coefficient by 90% and 76%, respectively. While the negative effect of CNT on the friction coefficient, an increase from 0.21 to 0.32 was observed. Naddaf et al.^39^ studied the tribological performance of diesel oil containing graphene and MWCNT as lubricating additives. Their results indicated that the heat transfer properties of nanofluids improved compared to the base oil. Esfe et al.^40^ reported that increasing MWCNT and TiO_2_ nanoparticles to engine oil cause to increase the dynamic viscosity. Pourpasha et al.^41,42^ studied the tribological and the thermophysical performance of turbine meter oil containing MWCNT and TiO_2_ nanoparticles as additives. Their results indicated that tribological and thermophysical properties of nanofluids improved compared to the pure lubricant.

Based on the many investigations conducted based on the lubricating oils, wide-ranging investigations have been led to lubricants, in contrast, no research has been done on turbine meter oil except for two articles published on it lately^41,42^. Turbine meters are used at gas pressure reducing and metering stations to measure gas volume. In this system, turbine meter oil is used to reduce wear and friction to the parts of the turbine meter. The primary purpose of this study is the numerically and experimentally evaluate the impacts of MWCNTs and TiO_2_ nano additives on the tribological and thermophysical properties of turbine meter oil. To achieve this goal, the first nano lubricants containing MWCNTs and TiO_2_ nanoparticles in various mass fractions (0.05 wt.%, 0.1 wt.%, 0.2 wt.%, 0.3 wt.%, and 0.4 wt.%) were synthesized through a two-step method. Then, the effect of the nanoparticle concentration on thermophysical and tribological features, including pressure drop, average friction coefficient, pour point, flash point, kinematic viscosity, relative viscosity, viscosity index, and wear, were studied. Some parts of the present study used the previously published papers data’s^41,42^ (Morphological analysis of MWCNTs, viscosity, viscosity index, flash point, and pressure drop data were used for MWCNTs/ turbine meter oil nanofluids^41^, also morphological analysis of TiO_2_ nanoparticles, viscosity, viscosity index, pour point, flash point, and wear data were used TiO_2_/turbine meter oil nanofluids^42^) in order to better compare the results of 2 different TiO_2_ nanoparticles and MWCNT nanotubes in turbine-meter oil rheological properties improving.

## Material and method

### Materials

MWCNTs and TiO_2_ nanoparticles as additives to improve the base fluid attributes were purchased from the VCN Materials Company and Sigma-Aldrich. The specifications of MWCNTs and TiO_2_ nanoparticles are shown in Table 1. Oleic acid and Triton x100 as the coating agents purchased from Merck. Table 2 shows the characteristics of Turbine meter oil as the base fluid that was purchased from Shell Oil Company.

**Table 1:** Nano additives characteristics.

Nanoparticles	Morphology	Diameter (nm)	Regular length (μm)	Purity (%)	Density (kg/m^3^)
MWCNTs	Tube shape	5–16.1	5–10	99	2100
TiO_2_	Spherical shape	7.9–13.9	–	99	4230

**Table 2:** Oil characteristics.

Fluid	Density (kg/m^3^)	Flash point (°C)	Pour point (°C)	Viscosity in 40 °C (cSt)	Viscosity in 100 °C (cSt)
Turbine meter oil	775	220	− 40	21.88	4.6

### Synthesis of nano lubricants

To prepare the TiO_2_/turbine meter oil, nano lubricants, a two-step method was used. Considering our previously published papers^41,42^ for this purpose, a certain amount of oleic acid (weight ratio of 1 to 2 with nanoparticles) as a surfactant and turbine meter oil were blended with the mixer at 1300 rpm for 30 min. Then, TiO_2_ nanoparticles were added to the solution and mixed for 4 h. Finally, to increase the stability and better distribution of additives in the pure lubricant, the prepared nano lubricants were exposed to ultrasonic waves for 3 h using an ultrasonic bath (Panasonic 2600 s). A similar method was used to synthesize nano lubricants containing MWCNTs, except that Triton x100 (weight ratio of 1 to 3 with nanoparticles) was used as a surfactant.

### Pressure drop measurement system description

**Figure 1:**
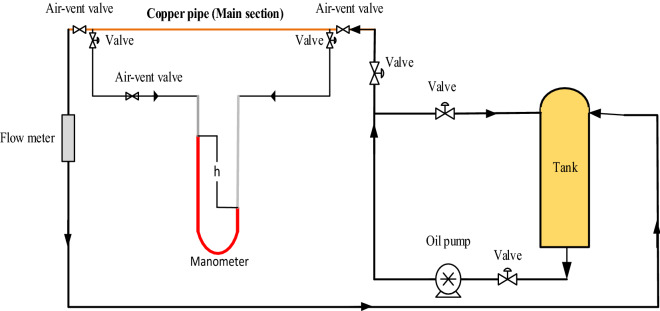
Schematic of the experimental system.

Figure 1 shows a schematic of the experimental system used to measure the friction coefficient and pressure drop of pure turbine meter oil and nano lubricant. The experimental system includes a manometer, oil tank, valves, pump, and decanter. This experiment was performed in different flow rates, and the following equation was used to calculate the flow rate (Q).

1 $$ {\text{Q}} = \frac{{\text{V}}}{{\text{t}}} $$

where v is a specific volume (ml) of circulating fluid in the system that comes out of the copper pipe at a constant time (t).

Equations (2) to (5) were used to calculate velocity (U), Reynolds for laminar flow (Re), friction coefficient (f), and the density of the nanofluid (${\uprho }_{{{\text{nf}}}}$), respectively.

2 $$ {\text{U}} = \frac{{\text{Q}}}{{\text{A}}} $$

where A is the cross-section of the copper tube.

3 $$ {\text{Re}} = \frac{{{\text{Ud}}}}{\upsilon } $$

In this equation, d is the copper pipe’s diameter, and $\upsilon$ is the viscosity of operating fluids.

4 $$ {\text{f}} = \frac{64}{{{\text{Re}}}} $$

5 $$ \uprho = \uprho_{{\text{n}}} \upphi + (1 - \upphi )\uprho_{{{\text{bf}}}} $$

where ${\uprho }_{n}$ is the density of nanoparticles, $\upphi$ is the particle volume fraction, and ${\uprho }_{bf}$ is the density of the base fluid.

The experimental pressure drop was measured through the manometer installed in the experimental system. For this purpose, water and CCl_4_, which are immiscible solvents, were used in the manometer, and the following equation was used to calculate the experimental pressure drop:

6 $$ \Delta {\text{P}}_{{{\text{exp}}}} = \uprho_{{{\text{CCl}}_{4} }} {\text{gh}}_{{{\text{CCl}}_{4} }} - \uprho_{{\text{w}}} {\text{gh}}_{{\text{w}}} $$

In this equation, ${\mathrm{h}}_{{\mathrm{CCl}}_{4}}$ and ${\uprho }_{{\mathrm{CCl}}_{4}}$ are the height and density of CCl_4_ in the manometer, ${h}_{w}$ and ${\uprho }_{w}$ are the height and density of water in the manometer, and g is the gravitational constant. Also, the theoretical pressure drop was calculated using Eqs. (7) and (8), respectively.

7 $$ \Delta {\text{P}}_{{{\text{theo}}}} = \frac{{{\text{fL}}\uprho {\text{U}}^{2} }}{{2{\text{d}}}} $$

where L is the length of the copper tube.

### Wear testing machine

**Figure 2:**
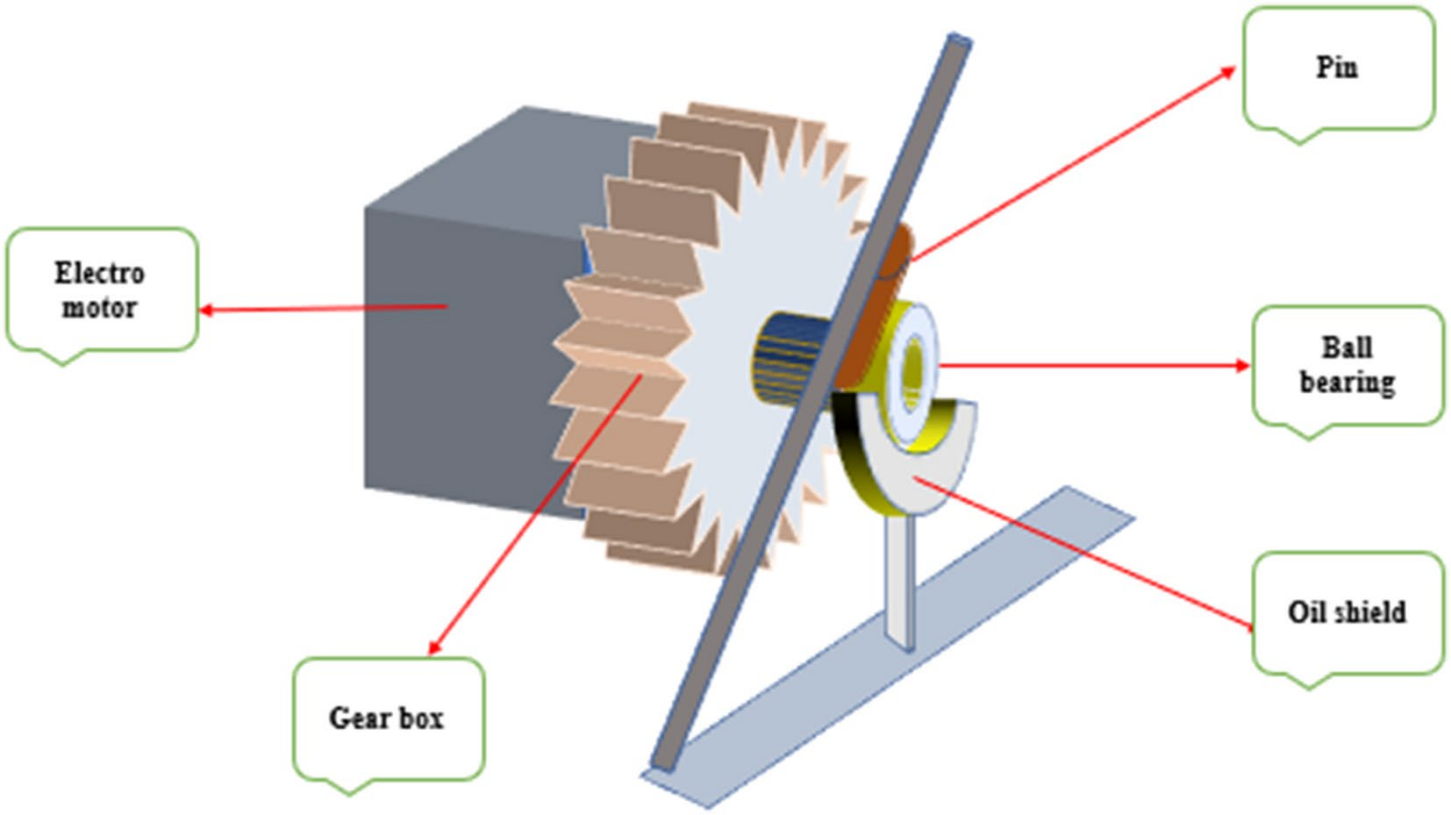
Schematic of the wear apparatus.

Figure 2 shows the schematic of the device designed to measure wear depth and coefficient of friction. This device includes a gearbox, electromotor, pin, oil shield, and ball bearing. All experiments were done under the same operating conditions, such as the load of 7 N, rotation at 200 rpm for 4 h, and the 60° angle between the pin and disc.

## Simulation description and numerical analysis

The pressure drops and the average friction coefficient inside the copper tube were calculated by continuity, momentum balance, and friction coefficient equations, as follows^41,43,44^:

8 $$ \frac{\partial \uprho }{{\partial {\text{t}}}} + \nabla \cdot \left( {\uprho {\vec{\text{v}}}} \right) = 0 $$

9 $$ \frac{\partial }{{\partial {\text{t}}}}\left( {\uprho {\vec{\text{v}}}} \right) + \nabla \cdot \left( {\uprho {{\vec{\text{v}}\vec{\text{v}}}}} \right) = - \nabla {\text{P}} + \nabla \tau + \uprho {\vec{\text{g}}} + {\vec{\text{F}}} $$

10 $$ {\text{f}} = \frac{{2{\text{d}} \Delta {\text{p}}}}{{{\text{L}}\uprho {\text{u}}^{2} }} $$

where $\mathrm{P}$, $\uprho \overrightarrow{\mathrm{g}},$ and $\overrightarrow{\mathrm{F}}$ are the static pressure, the gravitational body force, and external body force, respectively^41^. Utilizing boundary conditions and possible simplifications, the equations are solved, and the results are reported as a figure and table.

**Figure 3:**
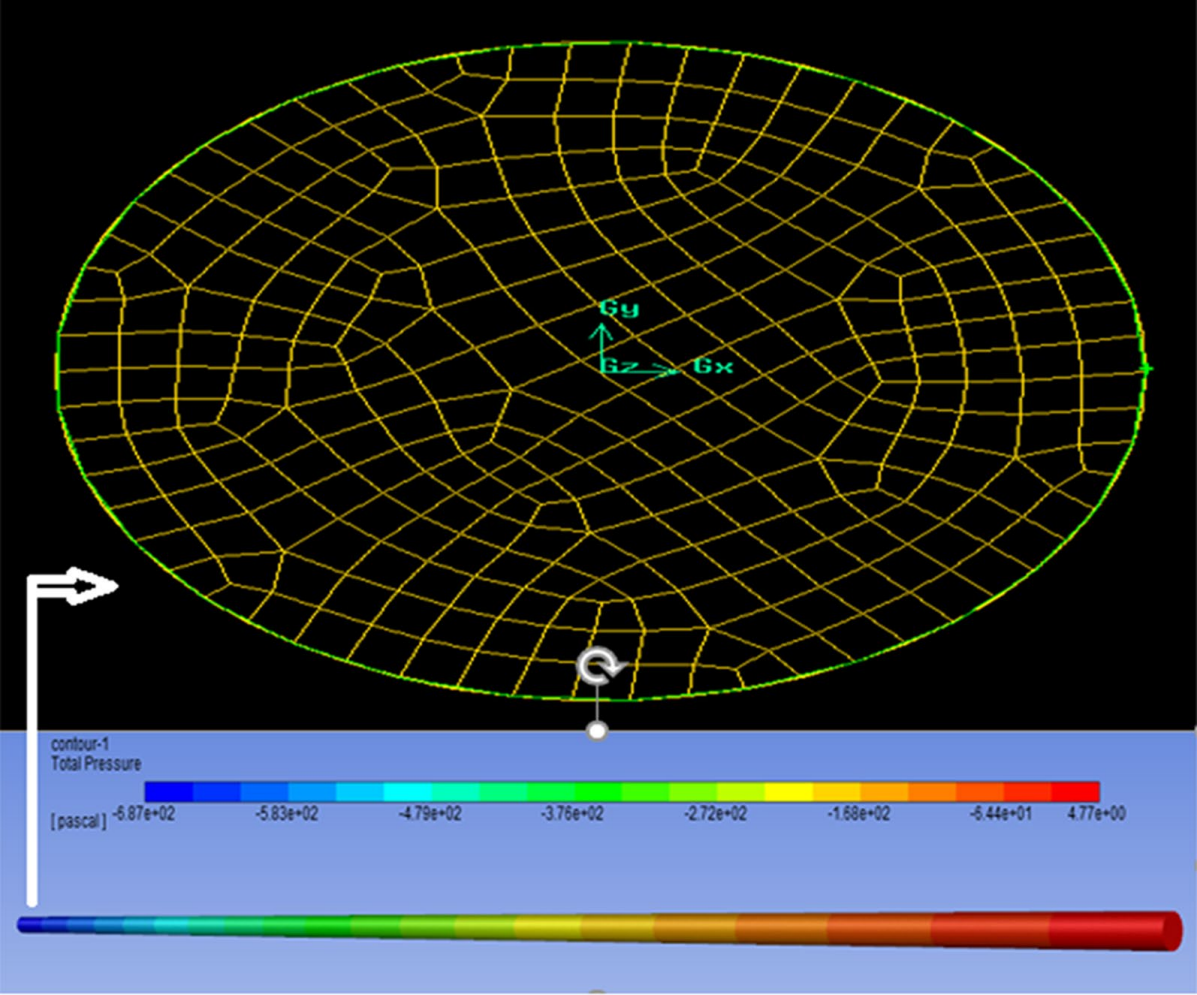
Schematic and mesh diagram of copper tube.

Boundary conditions play a necessary function in the computational fluid dynamics methods to enhance the convergence and validation of the resolution. The numeral figure of the computational domain of the pipe is indicated in Fig. 3.

The velocity inlet measuring range is between 0.069 m/s and 0.698 m/s, the copper pipe containing an interior diameter of 0.009 m and a length of 1.1 m. The attributes of the lubricant are imported into the materials section. The lateral configuration of the copper pipe has been considered a solid wall, the wall of the pipe has been weighed as an immovable wall, and no-slip boundary condition has been supposed in the wall of this tube. All of the inlet fluid exits the pipe, and the pump repeats this cycle until it attains a steady state.

The diagram of the copper pipe of the experimental setup has been designed and meshed by Gambit software, and the momentum balance and the friction coefficient equations have been solved by ANSYS-Fluent software based on the finite volume method. The mesh sizes of 1 mm was selected to simulate in this research work according to Table 3^41^, which represents a mesh independence analysis in three mesh sizes for lubricant as the working fluid with a velocity inlet of 0.069. The value of the friction coefficient and pressure drop are converged by decreasing the mesh size. Case 3 was selected as the optimum. The problem under study is a three-dimensional (symmetrical), steady, laminar flow of nano lubricant inside a copper pipe, and the pressure-based solving algorithm is appropriate. In the solution methods section of ANSYS-Fluent, the scheme, gradient, pressure, and momentum have been considered simple, least-squares cell-based, second-order, and second-order upwind, respectively. Convergence absolute criteria of continuity equations and velocity equations have been assumed with residual lower than 10^–13^.

**Table 3:** Mesh independence analysis in three different mesh sizes for turbine meter oil as the operating fluid with a velocity inlet of 0.069^41^.

Case	Mesh size (mm)	Number of elements	Aspect ratio	F	$\Delta P_{sim}$ (pa)
1	4	3509	2–4	3.0164	680.21
2	3	7110	1–4	3.0374	684.91
3	1	91,728	1–3	3.0396	685.39

## Results

In this part, the impact of adding TiO_2_ and MWCNTs nanoparticles on lubricant properties, including viscosity, viscosity index, flash point, pour point, wear, friction coefficient, and pressure drop, has been discussed in detail. Also, the impact of velocity inlet on the friction coefficient and pressure drop has been investigated.

### Characteristics analysis

**Figure 4:**
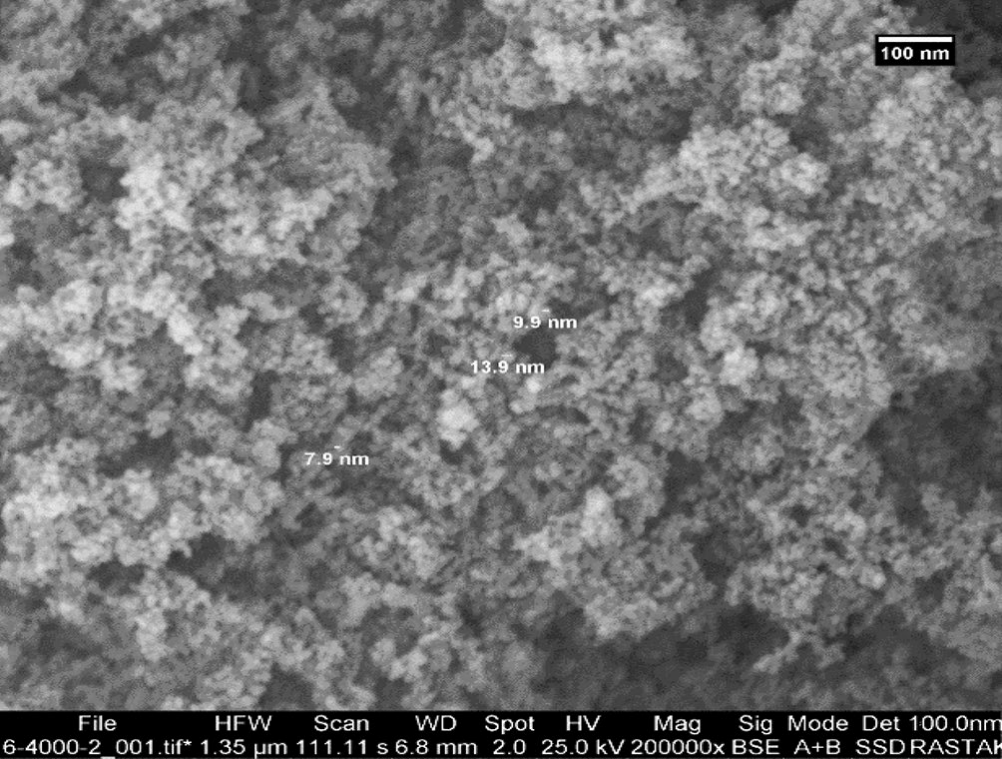
SEM images of the TiO_2_ nanoparticles^42^.

**Figure 5:**
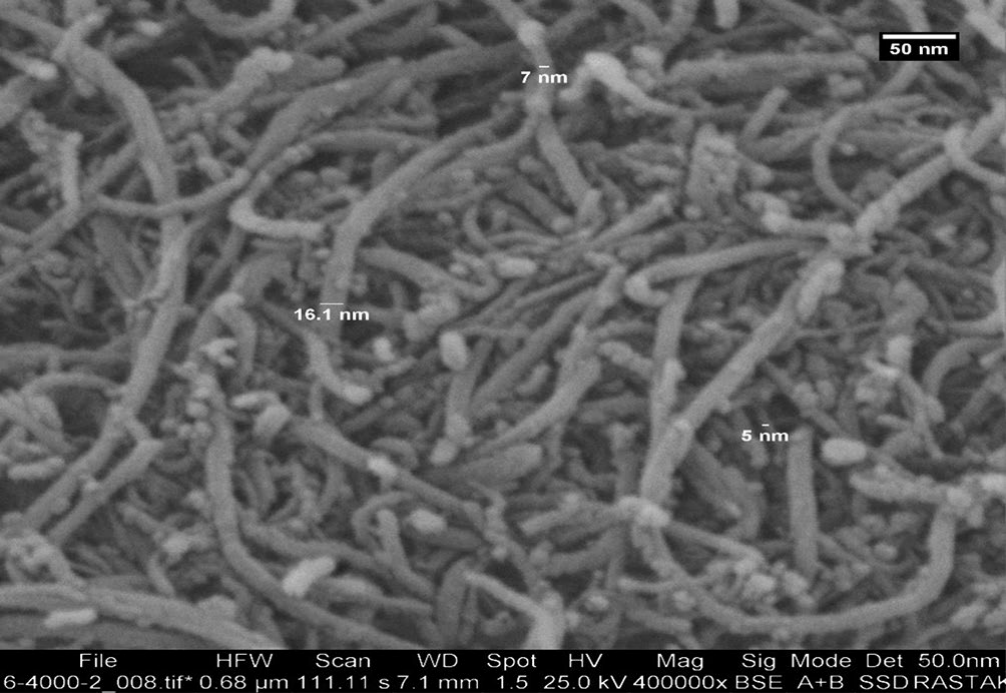
SEM images of the MWCNTs nanoparticles^41^.

Figures 4 and 5 indicate the SEM images of TiO_2_ and MWCNTs^41,42^. These SEM images were taken from TiO_2_ and MWCNTs at 200,000 and 400,000 magnifications, respectively. The diameter of the TiO_2_ nano additive is ranging from 7.9 to 13.9 nm, and the diameter of MWCNTs is ranging from 5 to 16.1 nm. Also, the TiO_2_ and MWCNTs additives are spherical shaped and tube shaped, respectively^41,42^. As can be seen, the TiO_2_ and MWCNTs have a uniform distribution range.

**Figure 6:**
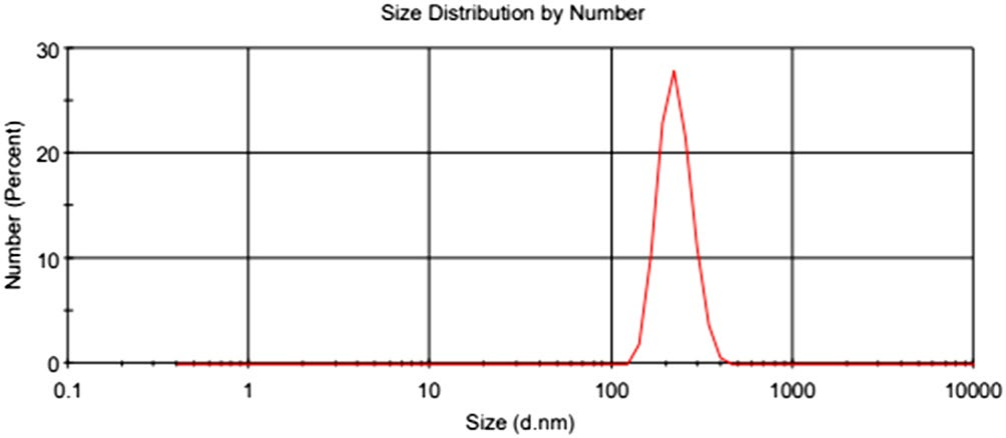
DLS images of the TiO_2_ nanoparticles.

**Figure 7:**
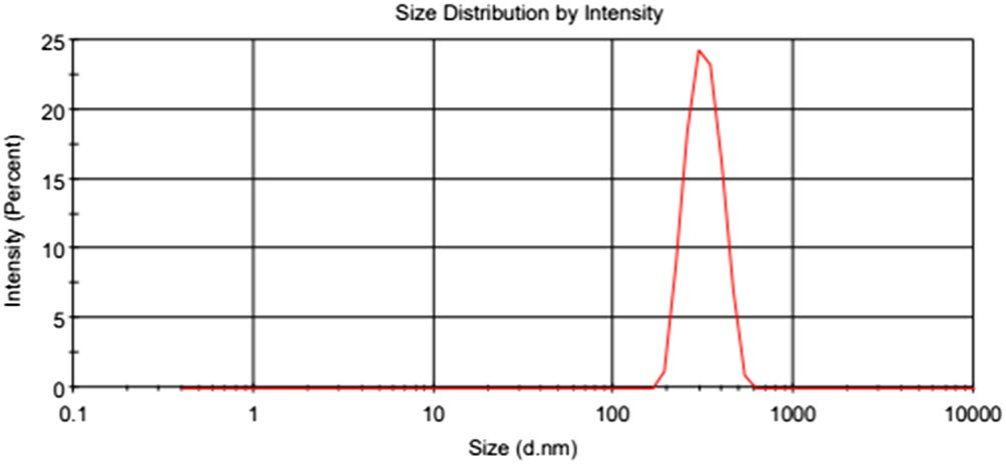
DLS images of the MWCNTs nanoparticles^41^.

According to Figs. 6 and 7, the size distribution of TiO_2_ and MWCNTs nanoparticles in 0.4 wt.% in the paraffinic base lubricant indicates that the average particle size of TiO_2_ and MWCNTs are 221 nm and 320 nm^41^, respectively. The mean diameter of the MWCNT in the pure oil was higher than TiO_2_ nanoparticles. According to research articles^45–47^, the addition of nano additives in the pure lubricant reason increases the size of the nano additives in the lubricant. Some critical parameters in the distribution of nano additive size in the pure fluid are the size, form of the nano additives, the type of pure fluid, and nano additives. If the proportion of the length to the diameter of nano additives is near to one, the form of the nano additives becomes near to spherical, and the average particle size is declined.

**Figure 8:**
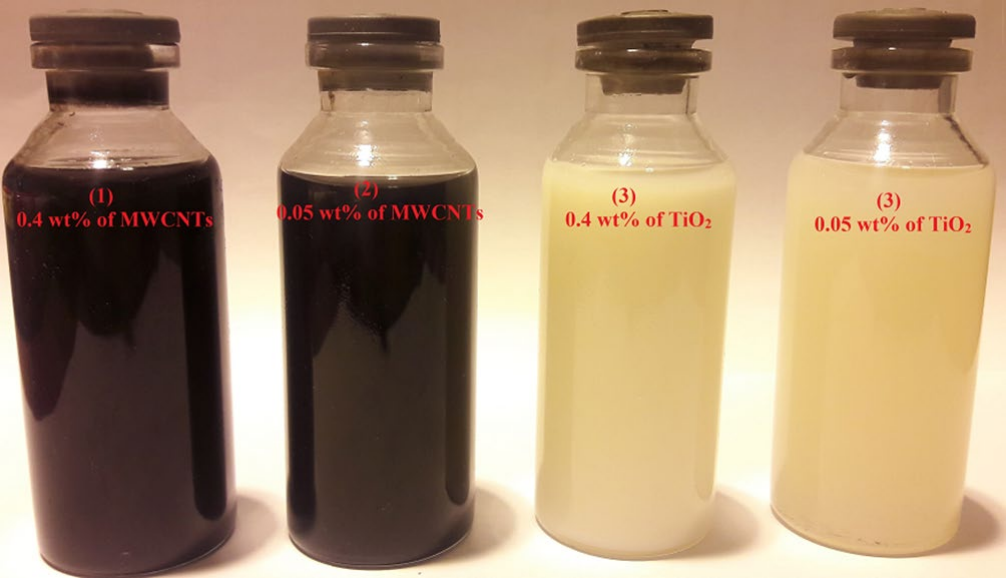
Camera pictures of nano lubricants after 2 h (1) 0.4 wt.% of MWCNTs, (2) 0.05 wt.% of MWCNTs, (3) 0.4 wt.% of TiO_2_, (4) 0.05 wt.% of TiO_2_.

To evaluate the stability of nanofluids, three types of surfactants, including Gum Arabic, Oleic acid, and Triton x100, in different weight ratios (1:1, 1:2, and 1:3), were used. According to visual observations, nanofluids containing MWCNTs using Triton x100 as a surfactant in a weight ratio of 1:3 compared to other surfactants have relatively better stability. Also, nanofluids containing TiO_2_ using Oleic acid as a surfactant in a weight ratio of 1:2 compared to other surfactants have a relatively better stability. Figures 8(1, 2) and 9(1, 2) illustrate the stability of MWCNTs nano lubricants at 0.4 wt.% and 0.05 wt.% that are taken at room temperature after 2 h and 6 days, respectively. Considering the figures, it is observed that the MWCNTs nano lubricants at 0.05 wt.% and 0.4 wt.% have good dispersion stability up to 8 h and 5 h, respectively and, no precipitations were observed. After 8 h and 5 h for MWCNTs nano lubricants at 0.05 wt.% and 0.4 wt.%, precipitations were started, respectively. Figures 8(3, 4) and 9(3, 4) illustrate the stability of TiO_2_ nano lubricants at 0.4 wt.% and 0.05 wt.% that are taken at room temperature after 2 h and 6 days, respectively. Considering the figures, it is observed that the TiO_2_ nano lubricants at 0.05 wt.% and 0.4 wt.% have good dispersion stability up to 4 days and 2 days, respectively and, no precipitations were observed. After 4 days and 2 days for TiO_2_ nano lubricants at 0.05 wt.% and 0.4 wt.%, precipitations were started, respectively.

**Figure 9:**
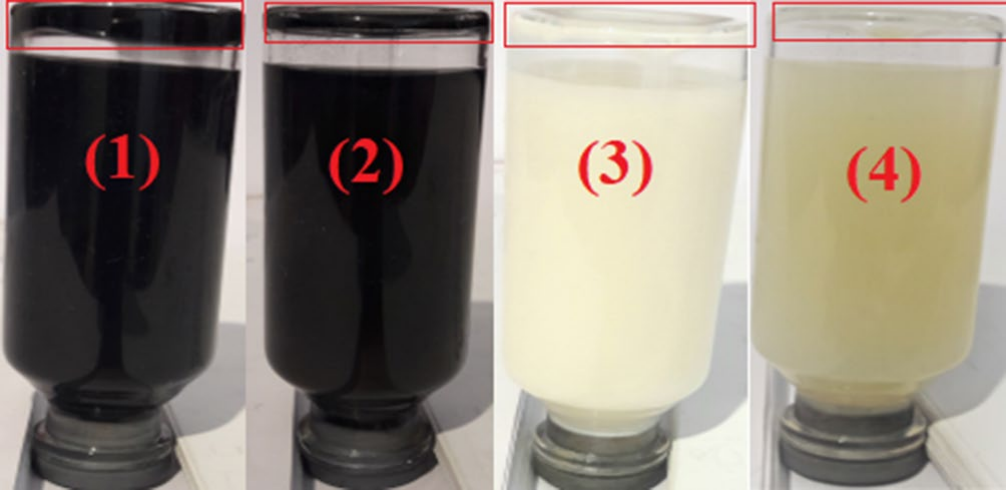
Camera pictures of nano lubricants after 6 days (1) 0.4 wt.% of MWCNTs, (2) 0.05 wt.% of MWCNTs, (3) 0.4 wt.% of TiO_2_, (4) 0.05 wt.% of TiO_2_.

**Figure 10:**
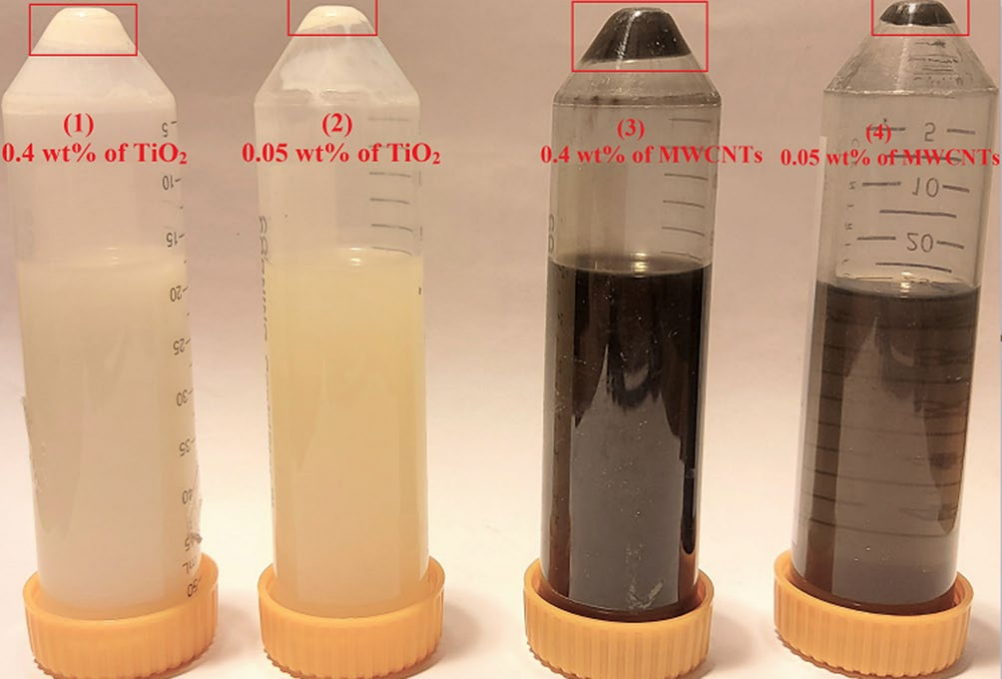
Camera pictures of nano lubricants after doing four steps of centrifugation (1) 0.4 wt.% of TiO_2_, (2) 0.05 wt.% of TiO_2_, (3) 0.4 wt.% of MWCNTs, (4) 0.05 wt.% of MWCNTs.

One of the methods for studying the stability of nanofluids is the centrifuge method. In this study, the stability of nanofluids containing MWCNTs and TiO_2_ nanoparticles in weight percentages of 0.4 wt.% and 0.05 wt.% by centrifugation in 1000, 2000, 3000, and 4000 rpm (revolutions per minute) were investigated. The centrifugation process is performed in 4 steps, including: (1) 40 min at 1000 rpm, (2) 10 min at 2000 rpm, (3) 10 min at 3000 rpm, and (4) 10 min at 4000 rpm. Figure 10(1, 2) illustrates the stability of TiO_2_ nano lubricants at 0.4 wt.% and 0.05 wt.%, after doing four steps of centrifugation. According to the test, after the third step for TiO_2_ nano lubricants at 0.05 wt.% and 0.4 wt.%, precipitations were started. Figure 10(3, 4) illustrates the stability of MWCNTs nano lubricants at 0.4 wt.% and 0.05 wt.%, after doing four steps of centrifugation. According to the test, after the two steps for MWCNTs nano lubricants at 0.05 wt.% and 0.4 wt.%, precipitations were started.

### Thermophysical analyses

Table 4 illustrates the kinematic viscosity changes of nano lubricants with the different mass fractions of TiO_2_ and MWCNTs nano additives at temperatures ranging from 30 to 100 °C. With increasing temperature from 30 to 100 °C, the kinematic viscosity of the nano lubricants and the base lubricant decreased. With increasing temperature, growth occurs in the Brownian motion of particles in the base lubricant. Hence, increasing the random rate of the particles declines the intermolecular forces among the pure fluid and the surface of the particles. The intermolecular forces of the lubricant are reduced with temperature rise and increasing molecular energy, which leads to an increment in the intermolecular distance. On the other hand, in the smaller sizes of nanoparticles and as the nanoparticle size decreases, the viscosity becomes much more dependent on the mass fraction of nanoparticles. As a result, the kinematic viscosity of nano fluids was declined with temperature rise^41,42^. With the addition of TiO_2_ and MWCNTs nano additives to the pure lubricant, the kinematic viscosity of the nano lubricants in the different mass fraction of nano additives relative to the pure lubricant enhanced at all temperature ranges. Thus, the maximum enhancement and the minimum enhancement of the kinematic viscosity (i.e., 6.83% and 2.59%, respectively) were obtained by increasing TiO_2_ nanoparticles to 0.4 wt.% at 30 °C and 60 °C, respectively. The maximum enhancement and the minimum enhancement of kinematic viscosity (i.e., 8.82% and 3.35%, respectively) were obtained with the addition of MWCNTs to 0.4 wt.% at 30 °C and 60 °C, respectively^41^.

**Table 4:** Comparison of the kinematic viscosity for TiO_2_/turbine meter oil and MWCNTs/turbine meter oil lubricants at different temperatures.

Kinematic Viscosity at various temperatures								
Concentration	30 °C	40 °C	50 °C	60 °C	70 °C	80 °C	90 °C	100 °C
Pure oil	29.95	20.849	15.158	11.243	8.599	6.762	5.472	4.501
0.05 wt.% MWCNTs	31.96	21.551	15.271	11.282	8.628	6.794	5.483	4.519
0.1 wt.% MWCNTs	31.942	21.651	15.408	11.418	8.754	6.907	5.583	4.624
0.2 wt.% MWCNTs	32.22	21.852	15.557	11.532	8.832	6.954	5.64	4.655
0.3 wt.% MWCNTs	32.593	22.071	15.693	11.62	8.903	7.019	5.67	4.677
0.4 wt.% MWCNTs	32.228	21.86	15.651	11.533	8.843	6.977	5.639	4.658
0.05 wt.% TiO_2_	30.982	21.232	15.192	11.342	8.736	6.818	5.599	4.624
0.1 wt.% TiO_2_	31.684	21.407	15.372	11.418	8.767	6.931	5.61	4.638
0.2 wt.% TiO_2_	31.768	21.645	15.313	11.418	8.797	6.969	5.659	4.676
0.3 wt.% TiO_2_	31.996	21.826	15.64	11.535	8.887	7.044	5.721	4.729
0.4 wt.% TiO_2_	31.996	21.826	15.64	11.535	8.887	7.044	5.721	4.729

**Figure 11:**
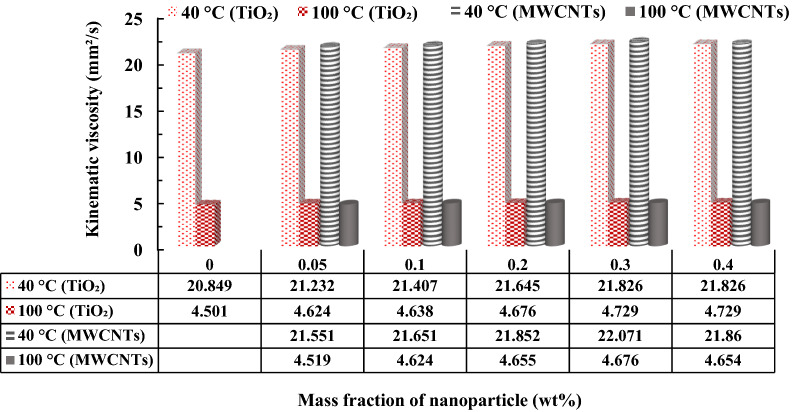
Comparison of the kinematic viscosity of TiO_2_/turbine meter oil and MWCNTs/turbine meter oil nanofluids in different mass fraction at 40 °C and 100 °C.

**Figure 12:**
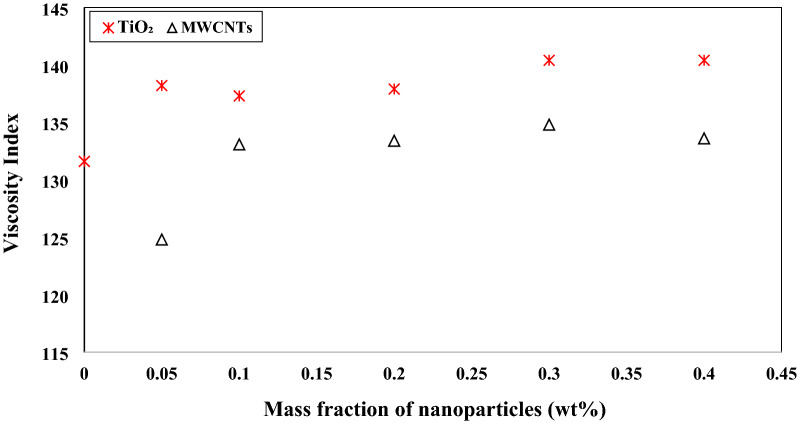
Comparison of viscosity index of TiO_2_/turbine meter oil and MWCNTs/turbine meter oil nanofluids.

The kinematic viscosity of TiO_2_/turbine meter oil and MWCNTs/turbine meter oil, nano lubricants in a different mass fractions at 40 °C and 100 °C, as shown in Fig. 11. The viscosity index was calculated using the kinematic viscosity values of base lubricant and nano lubricants at 40 °C and 100 °C, as shown in Fig. 12. The kinematic viscosity of the nanofluid with the addition of TiO_2_ nanoparticles at the mass fraction of 0.05–0.4 wt.% at 100 °C is higher than the kinematic viscosity of the nanofluid by increasing of MWCNTs with the same mass fraction. Consequently, nano lubricants with 0.3 wt.% of TiO_2_ and MWCNTs nano additives illustrated the maximum percentage of improvement in viscosity index with the enhancement of 6.68% and 2.43% compared to the pure lubricant, respectively.

**Figure 13:**
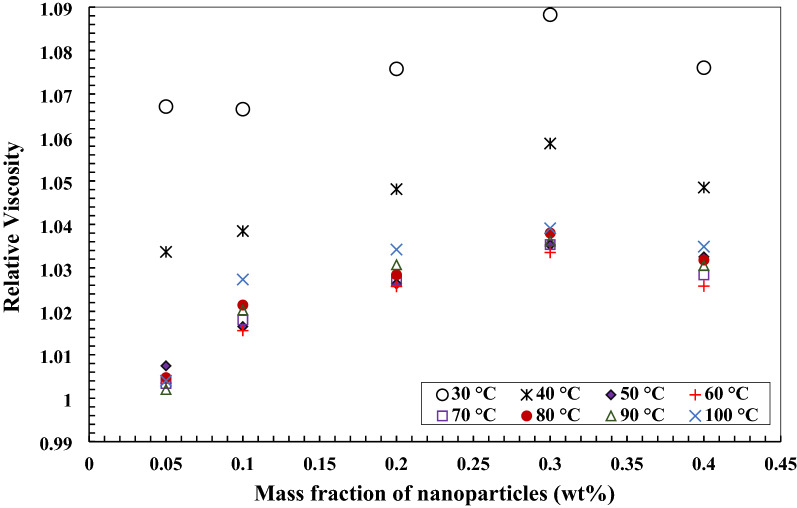
Variation of relative viscosity of MWCNTs/turbine meter nanofluids in different mass fraction.

**Figure 14:**
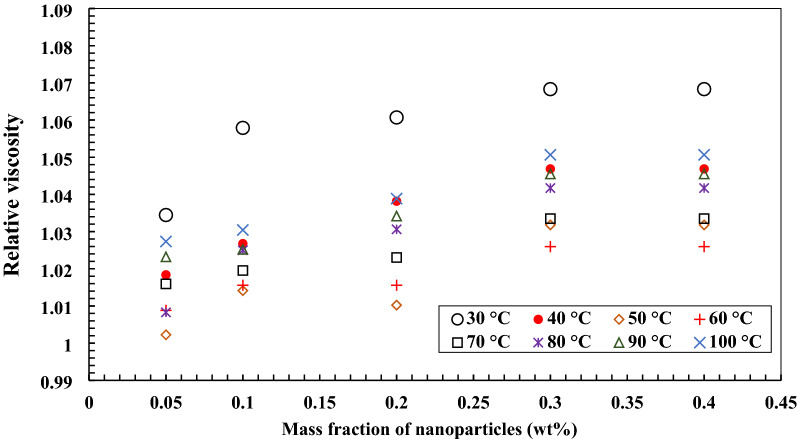
Variation of relative viscosity of TiO_2_/turbine meter nanofluids in different mass fraction.

Figures 13 and 14 illustrate the relative viscosity changes of the nano lubricant with the addition of TiO_2_ and MWCNTs nano additives in a mass fraction of 0.05 wt.%, 0.1 wt.%, 0.2 wt.%, 0.3 wt.%, and 0.4 wt.% of the temperature measuring range from 30 to 100 °C. By increasing the concentration of MWCNTs nanoparticles to base oil, the relative viscosity at the temperature measuring range increased. The same trend has been presented for nanofluids with TiO_2_ nanoparticles. The correlation for estimating the ratio of kinematic viscosity of MWCNTs/turbine meter oil nanofluid was obtained curve-fitting experimental data of nano lubricant temperature (°C) and MWCNTs mass fractions (wt.%). The following correlations were obtained for the temperature measuring range from 30 to 60 °C with R^2^ = 0.9795 and sum of squares = 2.278 × 10^–4^ (Eq. (11)) and the temperature measuring range from 70 °C to 100 °C with R^2^ = 0.9401 and sum of squares = 3.4 × 10^–3^ (Eq. (12)) for estimating the ratio of kinematic viscosity of nano lubricants. The correlation for estimating the kinematic viscosity ratio of TiO_2_/turbine meter oil nanofluid with 0.05, 0.1, 0.2, 0.3, and 0.4 wt.% of nanoparticles was obtained curve-fitting of experimental data of nano lubricant temperature (°C) and TiO_2_ mass fractions (wt.%). The following correlations were obtained for the temperature measuring range from 30 to 60 °C with R^2^ = 0.9419 and sum of squares = 5.4 × 10^–3^ (Eq. (13)) and the temperature measuring range from 70 °C to 100 °C with R^2^ = 0.9323 and sum of squares = 3.4 × 10^–3^ (Eq. (14)) for estimating the ratio of kinematic viscosity of nano lubricants.

11 $$ \upmu_{{\text{r}}} = \frac{{\upmu_{{{\text{nf}}}} }}{{\upmu_{{{\text{bf}}}} }} = 1.225 + 0.232{\text{w}} - 7.792 \times 10^{ - 3} {\text{T}} - 0.3672{\text{w}}^{2} + 6.617 \times 10^{ - 5} {\text{T}}^{2} $$

12 $$ \upmu_{{\text{r}}} = \frac{{\upmu_{{{\text{nf}}}} }}{{\upmu_{{{\text{bf}}}} }} = 1.0061 + 0.3066{\text{w}} - 5 \times 10^{ - 4} {\text{T}} - 0.5217{\text{w}}^{2} + 3.8376 \times 10^{ - 6} {\text{T}}^{2} $$

13 $$ \upmu_{{\text{r}}} = \frac{{\upmu_{{{\text{nf}}}} }}{{\upmu_{{{\text{bf}}}} }} = 1.18 + 0.1624{\text{w}} - 6.506 \times 10^{ - 3} {\text{T}} - 0.1973{\text{w}}^{2} + 5.717 \times 10^{ - 5} {\text{T}}^{2} $$

14 $$ \upmu_{{\text{r}}} = \frac{{\upmu_{{{\text{nf}}}} }}{{\upmu_{{{\text{bf}}}} }} = 0.9794 + 0.142{\text{w}} + 3 \times 10^{ - 4} {\text{T}} - 0.156{\text{w}}^{2} + 1.265 \times 10^{ - 6} {\text{T}}^{2} $$

**Figure 15:**
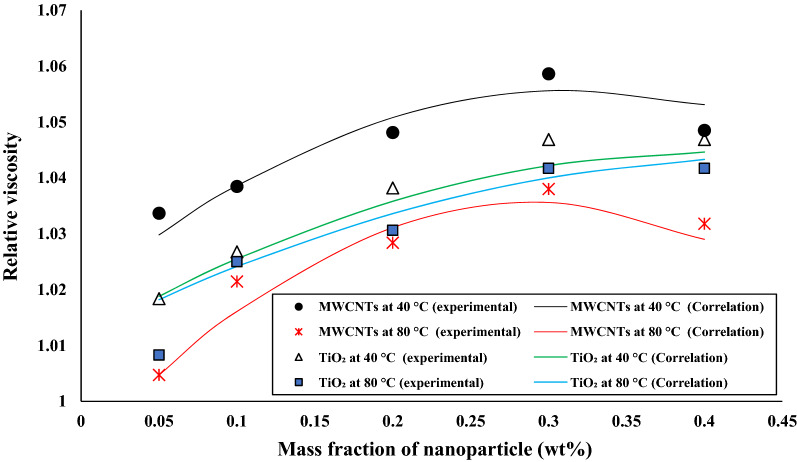
Variation of relative viscosity of TiO_2_/turbine meter and MWCNTs/turbine meter oil nanofluids in different mass fraction.

Figure 15 indicates experimental data and the correlation outputs from Eqs. (11), (12), (13), and (14) for the ratio of kinematic viscosity nanofluids with 0.05 wt.%, 0.1 wt.%, 0.2 wt.%, 0.3 wt.%, and 0.4 wt.% of TiO_2_ and MWCNTs nano additives at 40 °C and 80 °C. The correlation outputs and experimental data are closer together or show a minor deviation that calculation of Margin of deviation Eq. (15) indicate the maximum deviation margin is 1%.

15 $$ {\text{Margin}}\;{\text{of}}\;{\text{deviation }} = \frac{{\upmu_{{r_{Exp} }} - \upmu_{{r_{Correlation} }} }}{{\upmu_{{r_{Exp} }} }} $$

**Figure 16:**
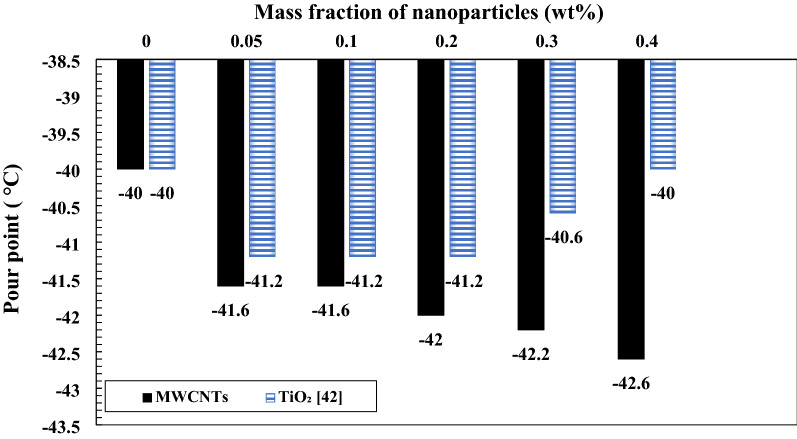
Comparison of pour point of TiO_2_/turbine meter oil and MWCNTs/turbine meter oil nanofluids in different mass fraction.

**Figure 17:**
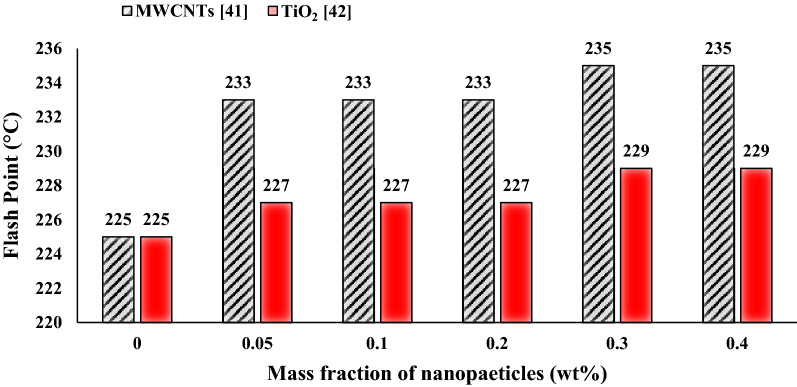
Comparison of flash point of TiO_2_/turbine meter oil and MWCNTs/turbine meter oil nanofluids in different mass fraction.

Figure 16 illustrates the impact of TiO_2_ and MWCNTs nano additives on the pour point of turbine meter oil. By increasing TiO_2_ and MWCNTs to the base fluid, the pour point temperature of nanofluids improved compared with base fluid. Consequently, nano lubricants with 0.1 wt.% of TiO_2_ and 0.4 wt.% of MWCNTs nano additives illustrate the maximum improvement in pour point temperature with the decrease of 1.2 °C and 2.6 °C compared to the pour point temperature of pure oil, respectively. Turbine meter oil has the lowest the pour point temperature among other lubricants. The addition of any additives, including nanoparticles and polymers, will have little effect on the pour point temperature of base oil, Due to the type of refining lubricant and additives used in the production of this oil, which prevents the growth of wax crystals up to − 40 °C. Figure 17 illustrates the impact of TiO_2_ and MWCNTs nano additives on the flash point of turbine meter oil. By increasing TiO_2_ and MWCNTs to the pure oil, the pour point temperature of nanofluids improved compared with base oil. Consequently, nano lubricants with 0.3 wt.% of TiO_2_ and 0.4 wt.% of MWCNTs nano additives illustrate the maximum improvement in the flash point temperature with enhancement 4 °C and 10 °C compared to the flash point temperature of pure oil, respectively. The addition of TiO_2_ and MWCNTs nano additives cause to improvement in the thermal conductivity of the lubricant and cause to delay in the evaporation of ignitable vapor cause to enhance the resistance of the pure oil to ignition.

### Tribological analyses

**Figure 18:**
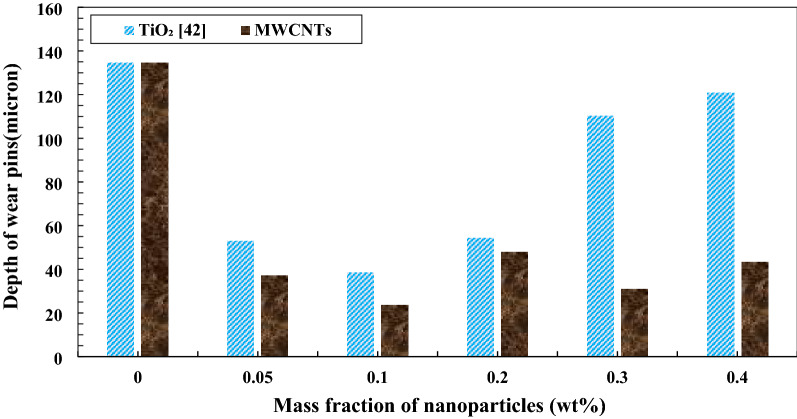
Comparison of the wear depth of copper pins with the addition of MWCNTs andTiO_2_ to the pure oil.

**Figure 19:**
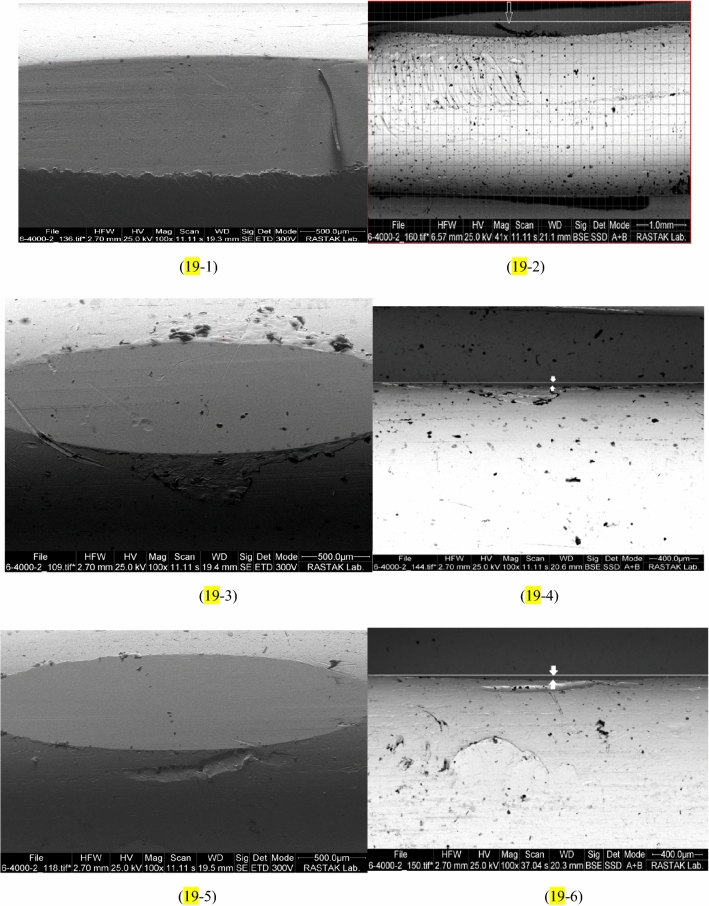

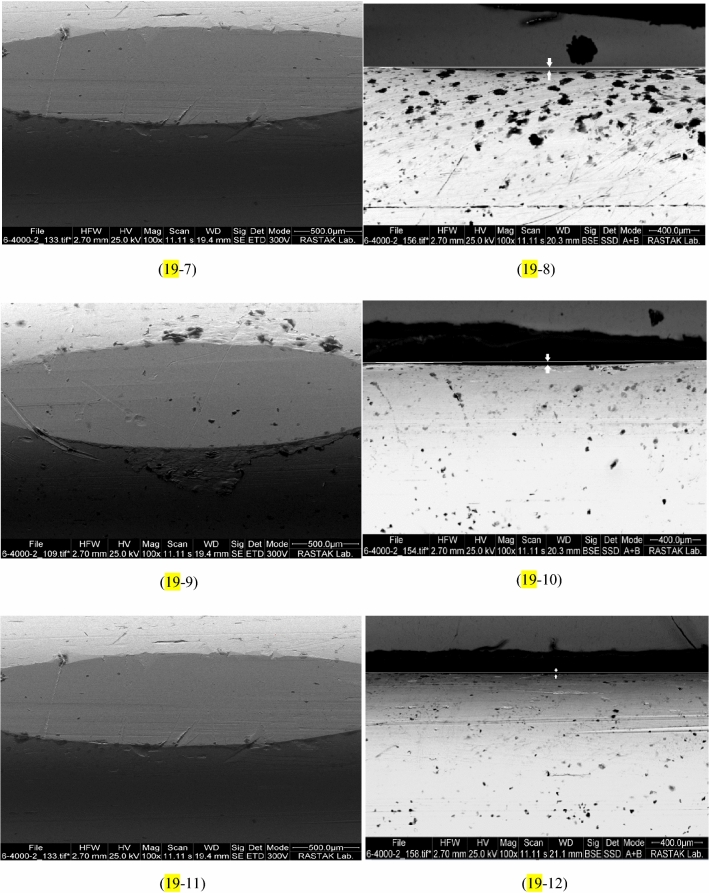
The wear depth of copper pins in the presence of: (**19-1**,**19-2**) pure fluid^42^, (**19-3**,**19-4**) nanofluid with 0.05 wt.% of MWCNTs, (**19-5**,**19-6**) nanofluid with 0.1 wt.% of MWCNTs, (**19-7**,**19-8**) nanofluid with 0.2 wt.% of MWCNTs, (**19-9**,**19-10**) nanofluid with 0.3 wt.% of MWCNTs, (**19-11**,**19-12**) nanofluid with 0.4 wt.% of MWCNTs.

The wear depth of copper pins was calculated by the SEM image, and the results of abrasion are reported in Fig. 18. By increasing MWCNTs nano additives to the base oil cause to decline in the wear depth of copper pins. The least abrasion or the most correction are related to 0.4 wt.% MWCNTs nano additives with a wear depth of 15.8 μm, compared to pure lubricant, it was bettered by 88.26%. SEM images of wear pins are shown in Fig. 19. The wear depth of the pins is indicated by a white line and two white arrows. Generally, the addition of MWCNTs nano additives to pure oil decreased the abrasion of pins. Also, Pourpasha et al.^42^ have presented the same trend for variations in the wear depth of copper pins by increasing TiO_2_ nano additives to the base oil. Their result, as shown in Fig. 18 with adding TiO_2_ nano additives to the pure oil, cause to reduce abrasion of pins that the least abrasion or the most correction are related to 0.1 wt.% TiO_2_ nano additives with a wear depth of 38.45 μm, compared to pure lubricant, was bettered by 71.43%^42^. The highest wear depth when using pure oil is 134.6 μm^42^. This suggests that nano-sized particle and based on morphology effectively play the roles of the following:

1. Formation of a uniform and stable film of nanofluids on ball-bearing surface.
2. MWCNTs and TiO_2_ nanoparticles with coating on rough surfaces, create a protective layer between two levels.
3. MWCNTs and TiO_2_ nanoparticles added to the lubricant have a mending impact so that they can compensate for the mass loss by sitting on the friction surfaces and filling the scratches, leading to a decrease in surface roughness.
4. MWCNTs and TiO_2_ nanoparticles act like rollers and ball bearings between two levels of friction and decrease the contact between pin and ball bearing surfaces.

According to the results, the wear depth of copper pins with increasing of MWCNTs to pure lubricant has been more improved compared with increasing of TiO_2_ nano additive to the base oil, because of their excellent high-temperature mechanical properties and low density as well as good wear and frictional properties. On the other hand, this is may be attributed to increased shear strength of the adsorbed oil on the surface of the ball bearing, and the mechanism of antiwear is attributed to the deposition of MWCNTs nanoparticles on the worn surface, which may decrease the shearing stress, thus improving the tribological properties^35,48^.

### Pressure drop and friction coefficient inside the copper tube

**Figure 20:**
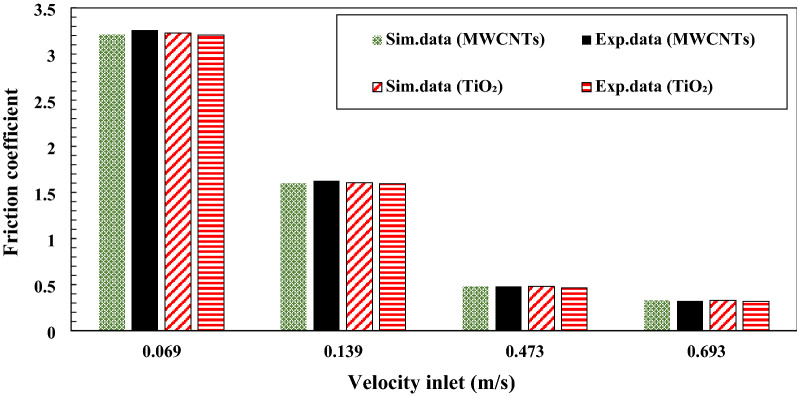
Comparison of experimental and simulation data of the average friction coefficient at the 0.05–0.4 wt.% of TiO_2_ and MWCNTs nano additives.

The average friction coefficient inside the copper tube with applying nanofluids as working fluids in the range of 0.05–0.4 wt.% of TiO_2_ and MWCNTs nano additives was calculated. Also, the variation of the average friction coefficient inside the copper tube with the various velocity inlet of nanofluids in the form experimental and simulation data are presented in Fig. 20. The simulation data and experimental data are closer together or show a minor deviation that the maximum deviation is 0.051. With increasing velocity inlet of lubricants led to an enhancement in the Reynolds number, which led to a decline in the average friction coefficient. The average friction coefficient inside the copper tube with using MWCNTs/turbine meter oil as a working fluid is a little more than the average friction coefficient of inside the copper tube with applying TiO_2_/turbine meter oil as a working fluid that the maximum difference between them is 0.053 at the velocity inlet of 0.069 m/s.

**Figure 21:**
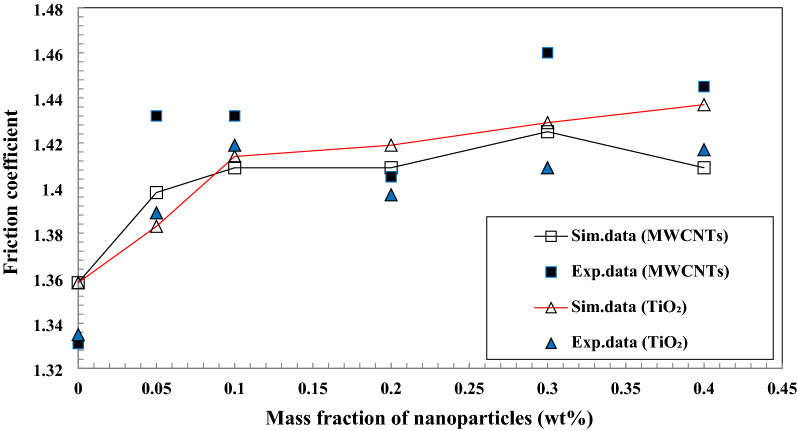
Comparison of experimental and simulation data of the average friction coefficient at the 0.069–0.698 m/s velocity inlet.

The average friction coefficient inside the copper tube with applying nanofluid as a working fluid in the range of 0.069–0.698 m/s velocity inlet of nanofluid was calculated. Also, the variation of the average friction coefficient inside the copper tube with the addition of TiO_2_ and MWCNTs nano additives with different mass fractions to pure oil that in the form experimental and simulation data are presented in Fig. 21. The simulation data and experimental data for this test are closer together or indicate a minor deviation that the maximum deviation is 0.036. The addition of the mass fraction of TiO_2_ and MWCNTs nano additives to the pure oil cause to increase in the average friction coefficient compared with the base oil. The maximum increase of the average friction coefficient (i.e., 9.36% and 6.14%, respectively) was obtained by increasing MWCNTs and TiO_2_ nanoparticles to 0.3 wt.% and 0.4 wt.% to the pure oil, respectively. Increasing concentration of nano additives cause to increases in the viscosity of nano lubricant and pressure drop, which increases the friction coefficient in the same conditions.

**Figure 22:**
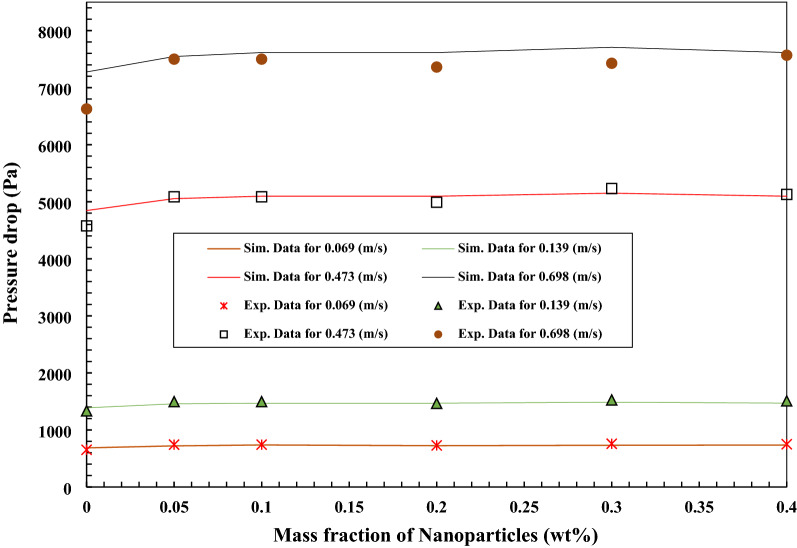
Comparison of experimental and simulation data of the pressure drop of the nanofluids in different mass fraction of MWCNTs nano additives.

**Figure 23:**
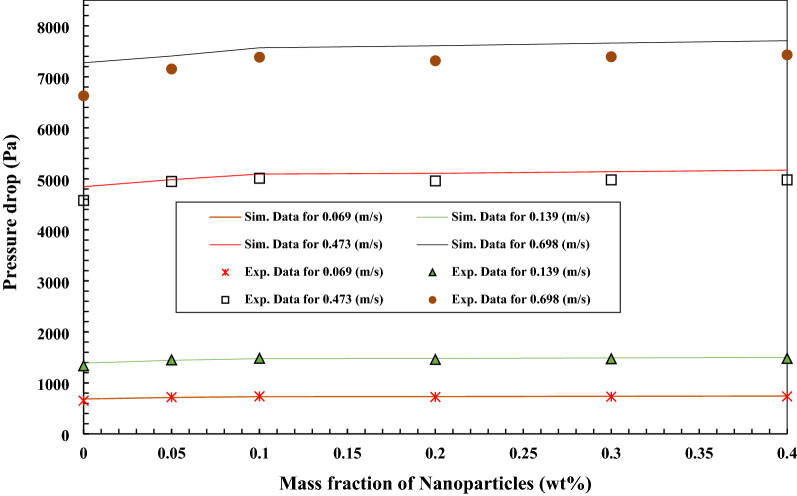
Comparison of experimental and simulation data of the pressure drop of the nanofluids in different mass fraction of TiO_2_ nano additives.

Figures 22 and 23 illustrate the simulation data and experimental data for the pressure drop changes of the nano lubricant with the addition of TiO_2_ and MWCNTs nano additives in the mass fraction of 0.05 wt.%, 0.1 wt.%, 0.2 wt.%, 0.3 wt.%, and 0.4 wt.% in the various velocity inlet boundary conditions. The simulation data and experimental data for the pressure drop are closer together and indicate a minor error that the maximum error is less than 10%. Increasing of MWCNTs and TiO_2_ to the base oil causes to increase in the pressure drop compared to the base oil. Also, the increasing of the velocity inlet increases the pressure drop compared to the pure lubricant, but the effect of increasing the velocity inlet of the nanofluids on the pressure drop is greater than the effect of adding nanoparticles to the pure fluids on the pressure drop. The maximum value of pressure drop of MWCNTs/turbine meter oil nanofluid with 0.3 wt.% of MWCNTs at the velocity inlet of 0.698 m/s was 7706.88 (Pa). The maximum value of pressure drop of TiO_2_/turbine meter oil nanofluid with 0.4 wt.% of TiO_2_ at the velocity inlet of 0.698 m/s was 7706.89 (Pa).

## Conclusion

The impact of MWCNT/turbine meter oil nano lubricant and TiO_2_/turbine meter oil nano lubricants with the different mass fraction of MWCNTs and TiO_2_ (0.05–0.4 wt%) and temperature measuring range from 30 to 100 °C on the average friction coefficient, pressure drop, pour point, flash point, relative viscosity, kinematic viscosity, viscosity index and average friction coefficient were investigated. Also, the pressure drop and the average friction coefficient inside the copper tube were simulated and compared with experimental results. From the obtained experimental and simulation results, the following conclusions are made:

1. With the addition of TiO_2_ and MWCNTs nano additives to the pure lubricant, the kinematic viscosity of the nano lubricants in different percentages of nano additives relative to the pure lubricant enhanced at all temperature ranges.
2. After 3 days and 2 days for TiO_2_ nano lubricants at 0.05 wt.% and 0.4 wt.%, precipitations were started, respectively. Also, after 8 h and 5 h for MWCNTs nano lubricants at 0.05 wt.% and 0.4 wt.%, precipitations were started, respectively.
3. Nanofluids containing MWCNTs using Triton x100 as a surfactant in a weight ratio of 1:3 compared to other surfactants have relatively better stability. Also, nanofluids containing TiO_2_ using Oleic acid as a surfactant in a weight ratio of 1:2 compared to other surfactants have relatively better stability.
4. The correlation outputs and experimental data of relative viscosity are closer together or show a minor deviation that calculation of Margin of deviation indicates the maximum deviation margin was 1%.
5. According to the results, the wear depth of copper pins with increasing of MWCNTs to pure lubricant has been improved compared with increasing of TiO_2_ nano additive to pure lubricant.
6. The average friction coefficient inside the copper tube with applying MWCNTs/turbine meter oil as a working fluid is a little more than the average friction coefficient of inside the copper tube with applying TiO_2_/turbine meter oil as a working fluid that the maximum difference between them is 0.053 at the velocity inlet of 0.069 m/s.
7. Nano lubricants with 0.1 wt.% of TiO_2_ and 0.4 wt.% of MWCNTs nano additives illustrated the maximum improvement in the pour point temperature with a decrease of 1.2 °C and 2.6 °C compared to the pour point temperature of pure oil, respectively.
8. Nano lubricants with 0.4 wt.% of TiO_2_ and 0.3 wt.% of MWCNTs nano additives illustrated the maximum percentage of increase the average friction coefficient with increasing of 6.14% and 9.36% compared to the pure lubricant, respectively.
9. The wear depth of copper pins with increasing of MWCNTs to pure lubricant has been more improved compared with increasing of TiO_2_ nano additive to the base oil, because of their excellent high-temperature mechanical properties and low density as well as good wear and frictional properties.

## References

[1] Huang J, Li Y, Jia X, Song H (2019). Preparation and tribological properties of core-shell Fe3O4@ C microspheres. Tribol. Int..

[2] Alves SM, Barros BS, Trajano MF, Ribeiro KSB, Moura E (2013). Tribological behavior of vegetable oil-based lubricants with nanoparticles of oxides in boundary lubrication conditions. Tribol. Int..

[3] Wu Y, Tsui W, Liu T (2007). Experimental analysis of tribological properties of lubricating oils with nanoparticle additives. Wear.

[4] Zhang R (2018). Well dispersive TiO_2_ nanoparticles as additives for improving the tribological performance of polyalphaolefin gel lubricant. Ind. Eng. Chem. Res..

[5] Wang B, Wang X, Lou W, Hao J (2010). Rheological and tribological properties of ionic liquid-based nanofluids containing functionalized multi-walled carbon nanotubes. J. Phys. Chem. C.

[6] Mousavi SB, Heris SZ, Hosseini MG (2019). Experimental investigation of MoS2/diesel oil nanofluid thermophysical and rheological properties. Int. Commun. Heat Mass Transf..

[7] Meng Y, Su F, Chen Y (2016). Supercritical fluid synthesis and tribological applications of silver nanoparticle-decorated graphene in engine oil nanofluid. Sci. Rep..

[8] Javadpour R, Heris SZ, Mohammadfam Y (2021). Optimizing the effect of concentration and flow rate of water/MWCNTs nanofluid on the performance of a forced draft cross-flow cooling tower. Energy.

[9] Almeida C (2020). Experimental studies on thermophysical and electrical properties of graphene-transformer oil nanofluid. Fluids.

[10] Akbarpour H, Rashidi A, Mirjalili M, Nazari A (2019). Comparison of the conductive properties of polyester/viscose fabric treated with Cu nanoparticle and MWCNT s. J. Nanostruct. Chem..

[11] Mousavi SB, Heris SZ (2020). Experimental investigation of ZnO nanoparticles effects on thermophysical and tribological properties of diesel oil. Int. J. Hydrogen Energy.

[12] AlTurki AM (2018). Low-temperature synthesis of core/shell of Co_3_O_4_@ ZnO nanoparticle characterization and dielectric properties. J. Nanostruct. Chem..

[13] Mohammadfam Y, Heris SZ, Khazini L (2020). Experimental investigation of Fe_3_O_4_/hydraulic oil magnetic nanofluids rheological properties and performance in the presence of magnetic field. Tribol. Int..

[14] Niste VB (2017). Self-lubricating Al-WS 2 composites for efficient and greener tribological parts. Sci. Rep..

[15] Maghsoudy N, Azar PA, Tehrani MS, Husain SW, Larijani K (2019). Biosynthesis of Ag and Fe nanoparticles using *Erodium cicutarium*; study, optimization, and modeling of the antibacterial properties using response surface methodology. J. Nanostruct. Chem..

[16] Kobayashi J, Kobayashi N, Itaya Y, Hasatani M (2019). Synthesis of Ni base nanoparticles by W/O emulsion combustion. J. Nanostruct. Chem..

[17] Lee K (2009). Understanding the role of nanoparticles in nano-oil lubrication. Tribol. Lett..

[18] Ghadimi A, Metselaar IH (2013). The influence of surfactant and ultrasonic processing on improvement of stability, thermal conductivity and viscosity of titania nanofluid. Exp. Thermal Fluid Sci..

[19] Kumar RS, Sharma T (2018). Stability and rheological properties of nanofluids stabilized by SiO_2_ nanoparticles and SiO_2_–TiO_2_ nanocomposites for oilfield applications. Colloids Surf. A.

[20] Han Z, Yang B, Qi Y, Cumings J (2011). Synthesis of low-melting-point metallic nanoparticles with an ultrasonic nanoemulsion method. Ultrasonics.

[21] Kao M-J, Lin C-R (2009). Evaluating the role of spherical titanium oxide nanoparticles in reducing friction between two pieces of cast iron. J. Alloy Compd..

[22] Sabareesh RK, Gobinath N, Sajith V, Das S, Sobhan C (2012). Application of TiO_2_ nanoparticles as a lubricant-additive for vapor compression refrigeration systems—An experimental investigation. Int. J. Refrigeration.

[23] Ingole S (2013). Tribological behavior of nano TiO_2_ as an additive in base oil. Wear.

[24] Ali MKA (2016). Improving the tribological characteristics of piston ring assembly in automotive engines using Al_2_O_3_ and TiO_2_ nanomaterials as nano-lubricant additives. Tribol. Int..

[25] Alghani W, Ab Karim MS, Bagheri S, Bagheri NAM, Gulzar M (2019). Enhancing the tribological behavior of lubricating oil by adding TiO_2_, graphene, and TiO_2_/graphene nanoparticles. Tribol. Trans..

[26] Hong FT, Schneider A, Sarathy SM (2020). Enhanced lubrication by core-shell TiO_2_ nanoparticles modified with gallic acid ester. Tribol. Int..

[27] Sharma V, Timmons RB, Erdemir A, Aswath PB (2019). Interaction of plasma functionalized TiO_2_ nanoparticles and ZDDP on friction and wear under boundary lubrication. Appl. Surf. Sci..

[28] Shariatzadeh B, Moradi O (2014). Surface functionalization of multiwalled carbon nanotubes with chitosan and magnesium oxide nanoparticles by microwave-assisted synthesis. Polym. Compos..

[29] Enayatpour B (2018). Adsorption kinetics of lysozyme on multi-walled carbon nanotubes and amino functionalized multi-walled carbon nanotubes from aqueous solution. J. Mol. Liq..

[30] Hoseyni S, Moradi O, Tahmacebi S (2013). Removal of COD from dairy wastewater by MWCNTs: Kinetics and thermodynamics. Fullerenes Nanotubes Carbon Nanostruct..

[31] Moradi O, Maleki M, Tahmasebi S (2013). Comparison between kinetics studies of protein adsorption by single-walled carbon nanotube and gold nanoparticles surfaces. Fullerenes Nanotubes Carbon Nanostruct..

[32] Vakili-Nezhaad G, Dorany A (2009). Investigation of the effect of multiwalled carbon nanotubes on the viscosity index of lube oil cuts. Chem. Eng. Commun..

[33] Bhaumik S, Prabhu S, Singh KJ (2014). Analysis of tribological behavior of carbon nanotube based industrial mineral gear oil 250 cSt viscosity. Adv. Tribol..

[34] Cornelio JAC, Cuervo PA, Hoyos-Palacio LM, Lara-Romero J, Toro A (2016). Tribological properties of carbon nanotubes as lubricant additive in oil and water for a wheel–rail system. J. Market. Res..

[35] Khalil W, Mohamed A, Bayoumi M, Osman T (2016). Tribological properties of dispersed carbon nanotubes in lubricant. Fullerenes Nanotubes Carbon Nanostruct..

[36] Salah N, Abdel-Wahab MS, Alshahrie A, Alharbi ND, Khan ZH (2017). Carbon nanotubes of oil fly ash as lubricant additives for different base oils and their tribology performance. RSC Adv..

[37] Gao T (2019). Dispersing mechanism and tribological performance of vegetable oil-based CNT nanofluids with different surfactants. Tribol. Int..

[38] Li X, Xu X, Zhou Y, Lee K-R, Wang A (2019). Insights into friction dependence of carbon nanoparticles as oil-based lubricant additive at amorphous carbon interface. Carbon.

[39] Naddaf A, Heris SZ, Pouladi B (2019). An experimental study on heat transfer performance and pressure drop of nanofluids using graphene and multi-walled carbon nanotubes based on diesel oil. Powder Technol..

[40] Esfe MH, Arani AAA, Madadi MR, Alirezaie A (2018). A study on rheological characteristics of hybrid nano-lubricants containing MWCNT-TiO_2_ nanoparticles. J. Mol. Liq..

[41] Pourpasha H, Heris SZ, Mahian O, Wongwises S (2020). The effect of multi-wall carbon nanotubes/turbine meter oil nanofluid concentration on the thermophysical properties of lubricants. Powder Technol..

[42] Pourpasha H, Heris SZ, Asadi A (2019). Experimental investigation of nano-TiO_2_/turbine meter oil nanofluid. J. Therm. Anal. Calorim..

[43] Farshchi ME, Aghdasinia H, Khataee A (2019). Heterogeneous Fenton reaction for elimination of Acid Yellow 36 in both fluidized-bed and stirred-tank reactors: Computational fluid dynamics versus experiments. Water Res..

[44] Pourpasha H, Mohammadfam Y, Khani L, Mohammadpourfard M, Heris SZ (2020). Thermodynamic and thermoeconomic analyses of a new dual-loop organic Rankine-Generator absorber heat exchanger power and cooling cogeneration system. Energy Convers. Manag..

[45] Hwang Y (2011). Effect of the size and morphology of particles dispersed in nano-oil on friction performance between rotating discs. J. Mech. Sci. Technol..

[46] Rehman WU (2019). Synthesis, characterization, stability and thermal conductivity of multi-walled carbon nanotubes (MWCNTs) and eco-friendly jatropha seed oil based nanofluid: An experimental investigation and modeling approach. J. Mol. Liq..

[47] Esfahani MR, Languri EM, Nunna MR (2016). Effect of particle size and viscosity on thermal conductivity enhancement of graphene oxide nanofluid. Int. Commun. Heat Mass Transf..

[48] Lim D-S, An J-W, Lee HJ (2002). Effect of carbon nanotube addition on the tribological behavior of carbon/carbon composites. Wear.

